# Development of an Aboriginal Resilience and Recovery Questionnaire – a collaboration between practitioners and help-seeking clients of a Victorian Aboriginal community controlled health service

**DOI:** 10.1186/s12874-023-02091-4

**Published:** 2023-12-08

**Authors:** Graham Gee, Carol Hulbert, Helen Kennedy, Joanne Dwyer, John Egan, Linda Holmes, Anita Mobourne, Yin Paradies

**Affiliations:** 1 Intergenerational Health Group, Murdoch Children’s Research Institute, Parkville, VIC Australia; 2 School of Psychological Sciences, University of Melbourne, Parkville, VIC Australia; 3 Victorian Aboriginal Health Service, Fitzroy, VIC Australia; 4 Department of Paediatrics, University of Melbourne, Parkville, VIC Australia; 5 Victorian Aboriginal Child Care Agency, Preston, VIC Australia; 6 School of Humanities and Social Science, Faculty of Arts and Education, Deakin University, Burwood, VIC Australia

**Keywords:** Aboriginal, Torres Strait Islander, Resilience, Recovery, Trauma, Assessment

## Abstract

**Background:**

Indigenous experiences and perspectives of resilience, healing and recovery from trauma is gaining increasing attention, with a growing qualitative literature that spans multiple indigenous cultural groups. However, few quantitative measures are available. In this article, development of a preliminary version of the Aboriginal Resilience and Recovery Questionnaire is described.

**Aim:**

The first aim of this study was to describe findings from two focus groups that provided theoretical knowledge and development of items for a draft version of an Aboriginal Resilience Recovery Questionnaire. The second aim of the study was to conduct a preliminary psychometric analysis of the properties of the measure.

**Design:**

Multi-method research design grounded in indigenous research methodologies.

**Measures:**

Aboriginal Resilience and Recovery Questionnaire, Australian Aboriginal Version of the Harvard Trauma Questionnaire Trauma symptom subscale, Growth and Empowerment Measure.

**Results:**

(1) Two focus groups with six counselling staff from an Aboriginal health service were run that explored Victorian Aboriginal understandings of resilience, healing, and recovery from trauma. Sixty different protective factors viewed as potentially important to resilience, healing and recovery from trauma were identified by participants. (2) Following a review of the resilience literature, 75 items were reviewed and revised, with additional items developed by the focus group. (3) The final outcome was 60 items selected for a preliminary version of the Aboriginal Resilience Recovery Questionnaire, 50 of which made up 19 different subscales in addition to 10 single items. (4) Structured interviews were conducted with 81 help seeking Aboriginal clients recruited from the same health service. Preliminary psychometric assessment of the Aboriginal Resilience Recovery Questionnaire was undertaken using Principal Components Analysis. Two component subscales were extracted with adequate internal consistency and good convergent and discriminant validity. For both subscales there were moderate to strong positive associations with empowerment, and moderate to strong negative associations with trauma symptom severity.

**Conclusion:**

The preliminary results are promising for a strength-based resilience measure developed from the knowledge of Aboriginal practitioners and staff of a counselling service. Further research to address some psychometric limitations in the measure is required. A larger sample size will allow for a common factor analysis to be conducted. The Aboriginal Resilience Recovery Questionnaire has potential to assist Aboriginal Community Controlled Health Organisations and other organisations to evaluate whether services and programs can effectively support community members to strengthen individual, relational, community and cultural resilience resources.

**Supplementary Information:**

The online version contains supplementary material available at 10.1186/s12874-023-02091-4.

## Introduction

In recent years, indigenous^1^ First Peoples worldwide have begun to write about their experiences and understandings of resilience [1, 2]. Fleming and Ledogar [3] made the important point that understandings of resilience have existed in indigenous cultures millennia before the concept gained currency as a research construct. They identified multiple conceptual approaches to defining and operationalising resilience, such as resilience being viewed as an innate spiritual quality, or as a process involving the interplay between risk and protective factors. Recent critiques have also argued that resilience has been co-opted as a colonial discourse in which indigenous people are expected to adapt to oppression, marginalisation, and discrimination [4]. Arguably, these plurality of perspectives and research methodologies associated with indigenous resilience research is to be expected, and welcomed, given the diversity that exists within and between indigenous cultural groups.

In Australia, resilience has gained increasing attention within Aboriginal and Torres Strait Islander health research. Protective factors widely reported in the international resilience literature have been documented in studies involving Aboriginal and Torres Strait Islander peoples, such as participation in sports, achievement at school, social support, and self-esteem [5, 6]. Concurrently, much of this research has documented the protective effects of cultural determinants of wellbeing, such as experiencing belonging and connection to land, family and extended kinship systems, cultural practices, and connection to spirituality [7, 8]. These types of cultural determinants have been linked to a range of different health outcomes such as greater social and emotional wellbeing [9], positive mental health [10], and lower rates of chronic illnesses, such as diabetes and cardiovascular disease [11]. Similar constructions of resilience have emerged in research conducted with other indigenous First Peoples worldwide [12, 13].

Analyses of data from a national Aboriginal and Torres Strait Islander health survey in 2008 [14], found that, overall, strength of cultural attachment—defined as the extent to which respondents spoke an Indigenous language, recognised a traditional homeland, identified with a clan, tribal or language group, and participated in cultural activities—was positively associated with greater self-assessed health, higher employment, and lower levels of substance use [15]. Interestingly, while respondents reporting the highest level of cultural attachment reported the best health outcomes, those with intermediate levels of cultural attachment were more likely to report poorer outcomes in comparison to those who reported lower levels of cultural attachment. Other findings from the same survey revealed that moderate to strong levels of cultural attachment were associated with higher levels of psychological distress in comparison to respondents reporting lower levels of cultural attachment. Finally, for those living in urban regions, reporting a strong level of cultural attachment was more strongly associated with experiencing more racial discrimination in comparison to those reporting strong levels of cultural attachment in remote regions of Australia [16].

These findings are consistent with ecological understandings of resilience, where resilience is viewed as more than a signifier of individual capacities. Rather, it encompasses whole-of-system processes and mechanisms, including people’s capacity and opportunities to access resources and navigate systems, as well as structural level enablers or barriers that contribute to health and wellbeing outcomes in the face of significant adversity [17, 18]. One implication of this contextual-ecological understanding of resilience for Aboriginal and Torres Strait Islander mental health and social and emotional wellbeing research is that cultural determinants may operate differently depending on the types of adversity and health outcomes of interest. In addition, individual differences in the extent to which people draw on strengths and resources linked to cultural determinants of wellbeing are likely to influence the relationship(s) between adversity and wellbeing.

These intersections between individual differences in access to strengths and resources, resilience as a process including relationships and service-related resources, and the influential role of culture, has prompted researchers to investigate resilience across a wide range of cultures and cultural contexts [19–21]. One example is the International Resilience Project led by Ungar and colleagues which involved using mixed-method research approaches to develop a Child and Youth Resilience Measure (CYRM) [22, 23]. When investigating the meaning(s) of resilience for youth across 14 different communities and 11 countries, which included two First Nation Canadian cultural groups, they found that resilience was best defined as ‘both the capacity of individuals to *navigate* their way to the psychological, social, cultural, and physical resources that sustain their well-being, and their capacity individually and collectively to *negotiate* for these resources to be provided and experienced in culturally meaningful ways’ (p.225) [22]. When the team investigated the psychometric properties of a preliminary set of 58 resilience-related items, factor analysis of data gathered from 1451 youth revealed that while 28 items were found to be relevant to each geographical population group, there was also varying factor structures observed in response patterns across different locations (i.e., no single solution). Ungar and colleagues concluded that this reflected the ‘heterogeneity in how resilience is understood and negotiated across cultures and contexts’ (p. 142) [19].

Research findings in Australia and other countries have supported this understanding that resilience is contextual and shaped by culture. For example, a validation study of the 28-item CYRM in Canada involving 497 youth that used multiple social services found evidence for a three-factor solution [23], with subscales representing personal, relational, and contextual factors. Alternatively, a validation study of the same measure conducted in Aotearoa New Zealand that involved 593 youth currently engaged with the juvenile service system identified four factors, which included a social/cultural and spiritual/community subscale. In Australia, Langham and colleagues [20] investigated the psychometric properties of the short 28-item version of the CYRM among 233 Indigenous Australian boarding school students aged 11–17 years, from North Queensland communities. Using Exploratory and Confirmatory Factor Analysis, they found that their data did not fit previous findings for the CYRM-28 factor structure, and that the best fit comprised two scales, titled Sources and Expressions of Resilience. Robinson and colleagues [21] investigated the psychometric properties of a shorter version of the CYRM, comprising 12 items that represented ‘socio-cultural’ dimensions of resilience, in addition to using the Connor-Davidson Resilience Scale (CD-RISC 10) that represented individual psychological dimensions of resilience. Interviewing 520 Australia Aboriginal middle school students from three remote community schools in the Northern Territory, they found that although the construct validity of a single factor for both measures were a good fit, the dimensions of resilience were associated in different ways to life stressors and distress. For example, higher scores on the CD-RISC 10 measure of psychological resilience were positively associated with psychological distress, whereas higher CYRM-12 socio-cultural resilience scores were associated with lower psychological distress. Robinson and colleagues [21] concluded that dimensions of individual psychological resilience alone may be too narrow to capture the important types of social-cultural resources that remote community students drew upon to navigate and manage higher levels of adversity and distress. It is important to note that the studies briefly reviewed here involved youth and occurred several years after the study reported in this article. Also notable, there remains a current lack of corresponding resilience related research among Aboriginal and Torres Strait Islander adult help-seeking populations.

**Fig. 1:**
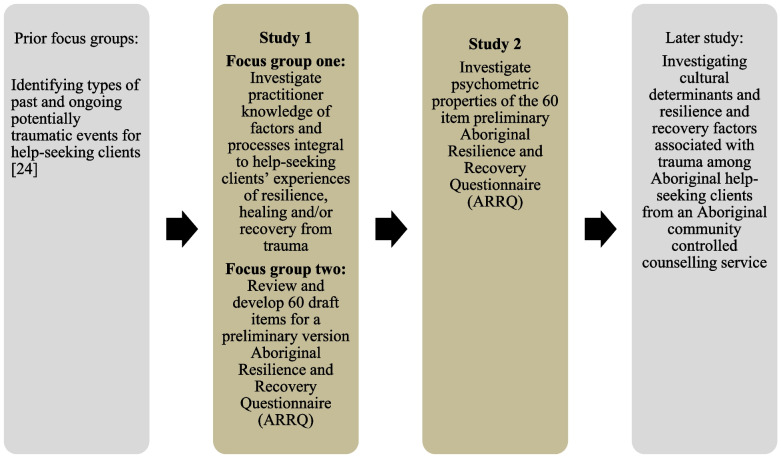
Flow chart showing Study 1 and 2 within larger program of research

These types of complex relationships between culture, resilience and mental health outcomes motivated a team of Aboriginal health professionals working at the Victorian Aboriginal Health Service (VAHS) in Melbourne, Australia, to investigate resilience, healing, and recovery from trauma among clients and community members using VAHS and its Family Counselling Services in 2011–2012. The VAHS is an Aboriginal Community Controlled Health Organisation established in 1973 by *Koori*^2^ leaders and Elders in recognition that existing health services had failed to meet the needs of the Victorian Aboriginal and Torres Strait Islander community. An exploration and review of the rich cultures of the Koori First Peoples of Victoria has highlighted how their resilience and resistance enabled their distinct cultures to continue to be transmitted and revitalised across generations. The review also highlighted the extent to which the Koori First Peoples of Victoria suffered brutal and rapid consequences of colonisation that included massacres, dispossession of land, and structural sanctioned violence through policies of child removal and assimilation [24]. Hence, the current situation for many Victorian Aboriginal and Torres Strait Islander peoples, families and communities includes both extraordinary resilience to survive and thrive despite intergenerational policies associated with assimilation, as well as significant social and health inequalities among those most vulnerable [25]. These findings contextualise the research findings reported here. This article describes the development of an Aboriginal Resilience and Recovery Questionnaire that involved collaboration between Senior Aboriginal staff, Aboriginal practitioners and Aboriginal help-seeking clients at the VAHS Family Counselling Service. Specifically, the aims of the study were to identify modifiable protective factors to inform development of a draft version of an Aboriginal Resilience and Recovery Questionnaire, and to undertake preliminary assessment of the psychometric properties of the measure. The two studies reported here (study one and two as outlined in the method section) represent two stages of research that were embedded within a larger PhD program of research that explored resilience, healing, and recovery from trauma among clients and community members using VAHS between 2011–2012. Figure 1 situates study one and two within the larger program of research that was conducted.

## Method

The study was undertaken in two stages. The first stage involved two focus groups with VAHS Family Counselling Service Aboriginal staff and a review of relevant literature to identify potential items for inclusion in an Aboriginal Resilience and Recovery Questionnaire (ARRQ). The first focus group explored Victorian Aboriginal understandings of resilience, healing, and recovery from trauma. The second involved staff reviewing and developing draft items for a preliminary version of the ARRQ. The second study involved conducting a Principal Components Analysis to analyse responses and the psychometric properties of the ARRQ.

The study was reviewed and approved by the University of Melbourne Behavioral and Social Sciences Human Ethics Subcommittee, the University of Melbourne Human Research Ethics Committee, the VAHS research subcommittee, and the VAHS Board of Directors in 2010. The study was grounded in indigenous research methodologies, drawing on three core principles of privileging Aboriginal and Torres Strait Islander voices, political integrity, and resistance as an emancipatory imperative [26]. This methodology ensured that the project upheld the VAHS ethos of Aboriginal and Torres Strait Islander self-determination, and framed project discussions with local Elders, Aboriginal and Torres Strait Islander staff and Koori community members on the VAHS research subcommittee about important issues such as the design of the study, Aboriginal leadership, and monitoring of clients personal and cultural safety throughout the project.

### Study one method

Two focus groups were conducted with six Victorian Aboriginal staff members from the VAHS Family Counselling Services. The participants were all long-standing members of the Melbourne Victorian Aboriginal community who worked closely with each other and were experienced in working with Aboriginal adults, children or families affected by trauma. Due to the small, close-knit nature of the community, demographic details and work-related roles of the staff members are not reported here for confidentiality purposes. In a focus group run previous to those reported in this study, the Victorian Aboriginal staff members were asked to describe the different types of traumatic events that some clients attending the service experienced in their lives. Multiple types of events and experiences were identified as potentially traumatic for clients presenting to the service, including historical trauma associated with the negative impact of colonisation and past government policies and practices; interpersonal trauma involving direct experiences of personal victimisation (e.g., physical abuse, sexual abuse, family violence); and discrimination-based trauma, characterised by traumatic stress related to experiences of racism and discrimination. All these types of trauma experiences were viewed as important areas of need with respect to healing and recovery for clients presenting at the service.

In Focus Group One, participants were asked to consider the types of protective factors and processes integral to help-seeking clients’ experiences of resilience, healing and/or recovery from trauma. Key questions included ‘what are some of the things that enable our clients and other people in the community to be resilient to, or recover from, trauma?’, ‘when you see clients and people in the community overcome traumatic experiences, how does this occur?’ and ‘what does recovery from trauma look like? Can you describe what you see when a client experiences trauma recovery?’.

Qualitative thematic analysis of the first focus group discussion was conducted to identify and organise factors and processes related to resilience, healing, and recovery from trauma. The raw interview data was hand coded according to topics and themes. The coding followed an iterative process of open coding, axial coding, and selective coding [27]. The codes, core themes, and the cultural appropriateness of the language used to describe the findings were checked with the participants following the analysis to ensure interpretations and conclusions were an accurate reflection of their views. After participants had identified factors and discussed processes associated with resilience, healing or recovery from trauma, a list of commonly cited strengths and protective factors from the resilience and recovery from trauma literature were presented to the group and they were asked to identify any other important protective factors that they deemed important.

Following focus group one, the first author reviewed all strengths and protective factors identified by participants and selected 19 modifiable protective factors (Table 5) deemed amenable to therapeutic change to be included in a preliminary draft version of the ARRQ. A literature search was conducted for psychological measures in the Aboriginal and non-Aboriginal peer-reviewed trauma recovery related literature that included any of the identified 19 protective factors. Fifteen measures that assessed one or more of these 19 protective factors were identified. Only one adult questionnaire with a resilience component that had been developed with an Aboriginal and Torres Strait Islander population was located, the Westerman Aboriginal Symptom Checklist Adults, developed by Dr. Tracey Westerman [28]. However, the measure was not available for free public use and documentation of its psychometric properties were not then available. Details of Westerman’s measure have subsequently been made available. Interested readers are referred to Bright [29] on Dr Westerman’s website: indigenouspsychservices.com. The first author reviewed the 15 identified measures and drafted 75 items as the basis for developing the preliminary version of the ARRQ.

In Focus Group Two, participants were asked to review the draft items and develop new assessment items that they considered important to the process of resilience, healing and/or recovery from trauma within a Koori and Victorian Aboriginal and Torres Strait Islander context. They were given complete freedom to develop new items, omit draft items, merge items, and/or significantly modify the language of any items as they saw fit. The intended outcome was a preliminary, multidimensional questionnaire of strength-based items developed by Aboriginal mental health professionals, with language structure and content deemed suitable for use with Aboriginal and Torres Strait Islander help-seeking clients. Table 1 summarises the different phases of tool development.

**Table 1:** Stages of developing the aboriginal resilience and recovery questionnaire

Focus Group One	Draft item development	Focus Group Two	Psychometric analyses
Identifying processes that enable Aboriginal help-seeking clients to experience healing and recovery from trauma Identification of 75 relevant strengths and protective factors	Review of Aboriginal and non-Aboriginal measures used in research about resilience, healing and recovery from trauma Development of 75 draft items for focus group participants	Reviewing draft items Modifying items Omitting items Developing new items	Structure interviews with 81 Aboriginal help-seeking clients Psychometric analyses of ARRQ structure

### Study one results

The first focus group explored processes integral to help-seeking clients’ experiences of resilience, healing, and recovery from trauma. Participants also identified specific types of strengths and protective factors relevant to these processes. The three themes that underpinned the development of the ARRQ are reported here.

### Conceptualising resilience, healing, and recovery from trauma

Participants identified three post trauma pathways. Resilience to trauma was viewed as the prior development of strengths that could help to buffer the impact of potentially traumatic experiences. Healing and recovery from trauma were viewed as processes that occurred after being impacted by potentially traumatic experiences. While this involved recovery and reductions in symptoms of distress, participants also emphasised that healing and recovery from trauma did not necessarily mean that the impacts of past traumatic events, for example emotional pain or grief, were entirely absent in someone’s life. Posttraumatic growth was identified and viewed as a process that involved developing new strengths and ways of being that were partly in response to, and shaped by, past traumatic experiences. Participants noted that clients could potentially experience all three pathways in relation to trauma.

### Healing and trauma recovery as a holistic and interconnected process

Although the group identified discrete risk and protective factors associated with healing and trauma recovery, they emphasised that it needed to be viewed as a complex process that involved multiple factors and personal, family, community, and nation-wide healing pathways. Healing and recovery were understood as processes that needed to occur in culturally safe spaces, and a whole of person-community-historical lens was required because healing from traumatic experiences often involved family, extended kin, and other parts of the community. It also involved the collective healing of Australia as a nation from the unresolved injustices and trauma of colonisation.

### Context and protective and vulnerability effects

Most of the protective factors considered were discussed within contexts of a continuum, in which the absence or lower magnitude of a protective factor was viewed to be associated with an increased vulnerability, whereas a greater access to the same protective factor was viewed as increasing the likelihood of positive health and wellbeing outcomes. However, some factors were identified as having both potential risk and protective status depending on the context within which the factor was considered. An example provided was academic success, which was viewed as having protective effects for Koori adolescents who had experienced childhood trauma. However, while academic success comes with healthy challenges and can enable resilience, participants observed that the cultural environment in which education occurred mattered, and in some contexts could also involve risks related to wellbeing. Participants observed that some local high schools included a large Koori student population, which often meant that students experienced the benefits of staying connected to cousins and extended kin (e.g., family resources of support). However, some of these same high schools also held lower expectations of Koori students, potentially undermining students’ motivation. Participants added that, conversely, while the private education system often provided a higher quality of education and teacher expectations that could help enable academic success, the often-low number of attending Koori students conferred a greater likelihood of experiencing cultural isolation, racism, and a loss of community connection.

### Protective factors associated with resilience, healing and/or recovery from trauma

When asked to identify factors that enabled clients to experience either resilience, healing or recovery from trauma, participants identified approximately 30 person-centred factors. With no prompting during the focus group session, participants also shifted focus and identified other factors, including relational factors (e.g., family, partners, friends, and mentors), community factors (e.g., community safety and community engagement), cultural factors (e.g., cultural practices, spirituality, and cultural identity), and broader societal factors (e.g., education in the broader community about local culture). The group identified an additional 20 protective factors associated with either relationships, community, cultural or societal contexts.

Following this open-ended discussion and exploration of protective factors, participants were presented with a list of other factors associated with resilience, healing and recovery from trauma that were derived from related Aboriginal and Torres Strait Islander and non-Indigenous literature [24]. Additional factors were identified by the group, giving a total of approximately 60 different protective factors viewed as potentially important to resilience, healing, and recovery from trauma. Although there are clear overlaps and the divisions arbitrary, these protective factors identified by participants in Table 2 are grouped into personal, relational, community, cultural, and broader societal domains.

**Table 2:** Focus group protective factors that support trauma recovery

**Participant**	**Personal Factors**
3	Time and the opportunity to grieve
3	Working/keeping busy (staying engaged in life)
6	Having a purpose
1	Helping others
1	Having the opportunity to tell one’s story
4	Understanding the nature of the trauma one has experienced
4	Acceptance of what has happened (without self-blame)
2	Being able to acknowledge the injustice of past trauma and one’s innocence
2	Finding different ways to respond to the pain (i.e., coping skills)
2	Self-awareness and questioning the self and past experiences
2	Recognising that as children we use whatever way we can to survive
6	Having a sense of control over one’s story
3	Experiencing positive emotions (to help build self-esteem and confidence)
2	Insight into nature of the trauma, how one may be contributing to maintaining difficulties, and taking responsibility for change
2	Having a sense of control of one’s life
6	Impulse control
2	Expression of emotions related to past trauma in a safe environment
4	Anger as strength when understood and expressed in a healthy way
5	Emotional intelligence (managing emotions, understanding the emotions of others, knowing one’s biggest triggers and those of significant others)
4	Optimism
2	Persistence
3	Bi-cultural skills
3	Humour
3	Being able to trust one’s self and others
3	Having meaning and purpose in life
6	Self-esteem
2	Understanding one’s strengths and weaknesses
1	Being able to adapt
6	Feeling safe
3	Good social skills
5	Forgiveness
2	Acknowledgment of injustice from another
4	Self-acceptance
1	Self-responsibility for one’s own happiness
5	Having a good education
**Participant**	**Relational factors**
1	Breaking the silence about having experienced trauma
4	Having family that love unconditionally, are forgiving and non-judgemental
4	Having a family who can provide insights and be supportive
4	Support and empathy from partners
6	Partner relationships that provide a new way of being/doing in relationship
1	Peer support
6	Mentors who believe in you and push you to extend yourself
**Participant**	**Community factors**
1	Using Aboriginal community-controlled health services
2	Rebuilding community relationships and sharing
1	Support from Aboriginal staff to help build clients level of safety and trust
1	Confidence increases when clients engage with Aboriginal staff whom they know may have overcome their own trauma
5	Re-establishing community connections and community engagement
5	Support from Aboriginal staff who understand the cultural and historical context of client’s trauma
2	Validation from one’s own community members
5	Being able to access cultural-centred group work (sharing one’s story/pain)
5	Sharing the healing journey together with other community members
**Participant**	**Cultural factors**
2	Engaging in cultural healing practices
2	Strong cultural identity
4	Going back to country
4	Spirituality when it relates to one’s identity
4	Culture pride about one’s identity
1	Elders teaching local history
**Participant**	**Societal factors**
1	Society providing cultural education in schools about local Aboriginal history
2	Society providing education about cultural diversity
5	Society breaking down cultural stereotypes
2	Having non-Aboriginal Australians work together with Aboriginal people
1	Social justice and acknowledgment

The focus group participants reviewed 75 items drafted by the first author that were based upon a review of items from a range of psychometric measures currently used in the field of resilience and trauma recovery, and further items added following analysis of the second focus group session. The participants developed new items, omitted items, merged items, and significantly modified the language of other items. Most items were modified, with only a few remaining close to the original wording. Approximately 15 Items were added to either better capture some of the pre-existing underlying constructs (i.e., relational support) or to assess newly introduced constructs that were deemed particularly relevant to Koori and Victorian Aboriginal understandings of resilience, healing, and recovery from trauma. Some examples of new items and associated constructs included were ‘I try to understand why things happen to me’ (i.e., insight), ‘I choose not to blame other people for the decisions I make (i.e., self-responsibility), ‘I am able to accept difficult things that have happened in my past’ (i.e., acceptance), ‘I have family that love me even when I muck up’ (i.e., family support), ‘When stressed I am able to take time to care for myself” (i.e., self-care) and ‘I participate in cultural practices that give me peace’ (i.e., cultural practices).

The outcome of this process was 60 items that represented a wide range of theoretically derived resilience constructs deemed important for Aboriginal help-seeking clients. Of the 60 items, 50 make up 19 subscales (i.e., each subscale a resilience construct) that consist of between 2–5 items each. The 19 subscales include community connection, community opportunity, communal mastery, cultural identity, self-worth/self-esteem, persistence, meaning, trust, self-awareness, self-responsibility, emotion regulation, positive emotions, role models, attachment, safety, social support, self-efficacy, personal mastery, and problem solving. The remaining 10 items are single-item questions that include resilience constructs such as the extent to which people view spirituality as a source of strength, participation in cultural practices, and bicultural skills. The relationship between these 19 subscales was investigated in a preliminary psychometric analysis in Study 2.

### Study two method

The structured interview schedule was piloted with five VAHS staff members to ensure that the measures, research protocols and interview process were adequate, and to allow for any adjustments to be made. After agreeing to be contacted about the study, participants were contacted by telephone or in person at the health service by the first author. If a client wished to participate, a time was agreed upon and interviews were conducted in the health service counseling rooms. Most interviews were conducted at a time just prior to the clients seeing their regular counselor, drug and alcohol worker, general practitioner, or psychiatrist. Clients were given the option of being interviewed in person or completing the measure as a pen-and-paper questionnaire. They could also choose to have an Aboriginal male or female interviewer.

The average time of completion for the structured interviews was 90 min (range 45–120 min). Post interview debriefing included providing refreshments, discussion of any follow up support needs if participants experienced any distress post-interview, and the opportunity for participant feedback. On completion, participants were given a 75-dollar shopping voucher as a gesture of appreciation for sharing their experiences and time.

### Participants

Structured interviews were conducted with 81Aboriginal help-seeking clients who used the VAHS Family Counselling Service during 2011. Sixty per cent of participants (*n* = 48) attended the counselling service on a weekly to monthly basis and received regular therapeutic support from a general medical practitioner, psychiatrist, counsellor, psychologist, drug and alcohol worker, or the service’s Aboriginal men’s and women’s group. The other 40 per cent (*n* = 33) were a diverse group of clients who regularly accessed services at the main site of the VAHS (i.e., the dental or medical unit), while their use of the counselling service varied. For example, some were clients who had previously used the service regularly but now only required psychiatric medication reviews a few times a year, while others were Aboriginal health workers who used the service on an as needs basis (i.e., a few times a year if in crisis). Thirty-nine per cent of participants (*n* = 32) elected to be interviewed in person, and 61 per cent (*n* = 49) elected to fill out the structured interview as a pen-and-paper questionnaire. VAHS provides services to the Aboriginal and Torres Strait Islander population living in the greater metropolitan area of Melbourne. However, help-seeking clients that use the VAHS Family Counselling Service (and VAHS more broadly) may come from a diverse range of Aboriginal and Torres Strait Islander cultural backgrounds (i.e., different clan groups and geographical locations in Australia). Accordingly, the structured interview with participants included demographic information related to age, gender, employment, financial security, education, and cultural identification. This included whether participants identified as Koori (Victorian clan groups), other Aboriginal and/or Torres Strait Islander cultural groups, or bi-cultural affiliations (identifying with both an Indigenous and non-Indigenous family or cultural backgrounds). Participant demographics are provided in Table 3 and described in the results).

**Table 3:** Participants demographics

	Total	Percentage
Number of participants	81	
Indigenous affiliation		
Aboriginal	67	83%
Torres Strait Islander	0	0
Aboriginal and Torres Strait Islander	0	0
Bi-cultural (identified with Indigenous and non- Indigenous heritages/family cultural backgrounds)	14	17%
Identified as Koori (born in Victoria)	68	84%
Non-Koori (born outside Victoria)	13	16%
Koori (Victorian Aboriginal) Clan Group Affiliations Non-Koori, non-Victorian Clan Group Affiliation	12 15	
Age (years), Mean (SD)	41 (SD 13)	
Sex		
Women	43	53%
Men	38	47%
Employment		
Yes	45	55%
No	36	45%
Financial security		
Enough money for basic living expenses		
Yes	49	60%
No	32	40%
Secondary (year 12 high school) and higher education		
Completed secondary schooling	18	22%
Completed a diploma/certificate qualification (e.g., TAFE level)	55	70%
Completed a higher education tertiary degree	5	6%

### Recruitment

Participants were recruited using two methods. First, a flyer was placed inside in the VAHS Family Counselling Services (FCS) waiting room, notifying clients of the project, and providing contact details for participation. Second, clients were recruited by FCS counselors, drug and alcohol workers, psychiatrists, general practitioners, and intake and outreach workers, all of whom had given prior consent to be involved in participant recruitment. FCS staff involved reviewed the measures and structured interview so that they were familiar with the interview process their clients would undertake. Staff were given a plain language statement for interested clients that outlined the project aims, time required for the interviews, and the availability of counselors’ post-interview. All project documents and staff invitations to clients made it explicit that one section of the interview would involve asking clients about the types of potentially traumatic events experienced in their life but would not involve asking any details about personal experiences. During the recruitment period, ongoing communication between the lead researcher and FCS staff about the project occurred via fortnightly team meetings. This allowed for continuous feedback and a team response to any potential concerns that emerged. Inclusion criteria for clients were to be over 18 years of age and assessed by respective client practitioner to be mentally and emotionally stable enough to undergo a 60–90-min interview about trauma, resilience, healing, and recovery without experiencing significant distress or exacerbating current mental health difficulties. For safety reasons, this excluded any clients currently experiencing acute distress or severe mental health problems. Accordingly, only a limited number of clients attending FCS in 2011 were asked to participate.

### Measures completed during the structured interview

#### The Aboriginal Resilience and Recovery Questionnaire (ARRQ)

The preliminary version of the ARRQ comprises of 60 self-report questions with a 5-point Likert scale response format: 1 = not at all, 2 = a little, 3 = somewhat, 4 = a fair bit, and 5 = a lot. The ARRQ includes 19 resilience constructs that are all subscales consisting of 2–5 item. In addition, there are 10 single-item questions, each representing a different resilience construct. The convergent and discriminant validity of the ARRQ was examined by reviewing correlations between the ARRQ subscales, the Australian Aboriginal Version of the Harvard Trauma Questionnaire trauma symptom subscale [30] and the Growth and Empowerment Measure [33].

### The Australian Aboriginal Version of the Harvard trauma questionnaire trauma symptom subscale [30]

The Australian Aboriginal Version of the Harvard Trauma Questionnaire is a 47-item measure adapted from the Harvard Trauma Questionnaire, a cross-cultural trauma inventory developed at the Indochinese psychiatric clinic at Harvard University [31]. In this study, the second subscale was used. It contains 30 items, 16 of which correspond to the PTSD symptom criteria in the Diagnostic and Statistical Manual of Mental Disorders III-R (DSM-III-R) [32], and 14 cultural idioms of distress, identified by C. Atkinson [30], that lie outside the DSM-III-R PTSD criteria. All 30 items are rated on 4-point Likert-scale (where 1 = not at all, 2 = a little bit, 3 = a fair bit, and 4 = a lot).

#### The Growth and Empowerment Measure [33]

The Growth and Empowerment Measure is an outcome measure designed to capture changes in dimensions of empowerment according to the experiences of Aboriginal and Torres Strait Islander people who had participated in the Family and Well Being Program. The six-scenario subscale was used, and Haswell and colleagues [33] reported that the subscale has strong internal consistency with a Cronbach’s alpha value of 0.85.

#### Psychometric analyses to determine suitability for a principal components analysis

Principal Components Analysis was selected as the statistical method for a preliminary analysis of the psychometric structure of the ARRQ. This procedure was chosen over more sophisticated common factor analysis methods such as Exploratory Factor Analysis because the latter requires a minimum participant to item ratio of 5:1 [34, 35]. The sample size in this study (*N* = 81) was far below this ratio, and therefore the study conditions for an Exploratory Factor Analysis were not met [36, 37]. Principal Components Analysis is more robust to small sample size numbers and allows for the reduction of data from a large set of variables to a reduced set of variables, called principal components, with as little loss of information as possible [38].

Four metrics were examined to determine the suitability of the data for conducting a Principal Components Analysis: (1) Examining the means and standard deviations of the 60 items to ensure there was variability in the frequency distributions of the data; (2) Conducting a reliability analysis of the sub-scales. Guttman’s Lambda coefficient [39] of internal consistency reliability was used here in preference to Cronbach’s Alpha coefficient [40] as it has been shown to be a better estimate of the true reliability [41]; (3) The Kaiser-Meyer Olkin [42] test to determine sampling adequacy (the proportion of variance among variables that may be common variance); and (4) Bartlett’s test of sphericity [43] to examine whether correlations between items were sufficiently large.

### Study two results

During 2011, 81 help-seeking Aboriginal clients that used the Family Counseling Service and broader services at the VAHS were recruited to participate in structured interviews.

### Participant demographics/representativeness of the study population

There are a range of important demographic and socio-cultural characteristics of the 81 participants in the study that provide context about the study population. First, forty-five per cent of participants reported being unemployed, whereas the national unemployment rate for Aboriginal and Torres Strait Islander people reported in the 2012 Australian Aboriginal and Torres Strait Islander Health Survey (AATSIHS) was 20.9 per cent [44]. Twenty-two per cent of participants in the study reported completing secondary schooling (year 12) in comparison to the 2012 AATSIHS completion rate of 45.7 per cent [44]. Forty per cent of participants reported not receiving enough money for basic living expenses, such as rent and food. In comparison, 2012–2013 Australian census data found that 22 per cent of Aboriginal and Torres Strait Islander people were living in a household that in the previous twelve months had run out of food [45]. Finally, there was significant cultural diversity among participants, including 12 Koori clan groups and 15 other clan groups across Australia. Taken together, these descriptive statistics indicate that on average, the participants in this study were more likely to report socioeconomic adversities related to unemployment and education in comparison to other Aboriginal and Torres Strait Islander peoples as reported in national data.

### Results of preliminary analysis of ARRQ items and subscales

The means and standard deviations of the 60 items were examined to assess for variability in the frequency distributions of the univariate data. As illustrated in Table 4, 55 of the 60 items had means between 2.89 and 4.20, and standard deviations that ranged from 1.02–1.46, suggesting enough variability for inclusion in a Principal Components Analysis. Of the remaining five items, four had high mean scores (items 14 self-responsibility, 20 compassion, 37 personal control, 46 accessing community support, and 58 cultural identity). However, these items also had reasonable variability with standard deviations ranging from 0.84—1.07. Two of the 60 items had high mean score and low variability (items 56 cultural pride and 57 cultural importance). Given this study represents the earliest stage of item development and taking into consideration the theoretical importance of these constructs in the Aboriginal literature on healing and recovery from trauma [46–48], these two items were retained for purpose of the preliminary analysis.

**Table 4:** Means and standard deviations of the 60 items in the Aboriginal Resilience and Recovery Questionnaire

Item	Mean	SD	Item	Mean	SD	Item	Mean	SD
1	3.34	1.20	21	3.36	1.31	41	3.51	1.11
2	3.10	1.24	22	3.26	1.43	42	3.39	1.45
3	3.30	1.35	23	3.65	1.20	43	3.06	1.35
4	3.49	1.18	24	4.00	1.07	44	3.23	1.46
5	3.72	1.20	25	3.86	1.39	45	3.99	1.11
6	4.20	1.02	26	4.15	1.19	46	4.45	0.84
7	3.29	1.33	27	3.39	1.28	47	4.06	1.03
8	3.33	1.10	28	3.74	1.24	48	3.96	1.11
9	2.89	1.24	29	3.74	1.20	49	3.74	1.19
10	3.91	1.05	30	3.23	1.36	50	3.18	1.15
11	3.82	1.04	31	4.00	1.35	51	3.58	1.27
12	3.52	1.30	32	3.43	1.46	52	3.86	1.16
13	3.87	1.21	33	3.89	1.42	53	3.31	1.38
14	4.25	0.97	34	3.60	1.14	54	3.59	1.29
15	3.80	1.19	35	3.55	1.15	55	3.54	1.38
16	3.11	1.37	36	3.26	1.29	56	4.85	0.42
17	2.96	1.24	37	4.21	1.07	57	4.86	0.38
18	3.36	1.12	38	3.49	1.14	58	4.54	0.82
19	3.05	1.23	39	3.39	1.25	59	3.95	1.23
20	4.33	0.91	40	3.59	1.09	60	2.99	1.44

Guttman’s Lambda (λ) [39] values for the 19 subscales were examined. As listed in Table 5, eight of the subscales had reliability values within the higher range of 0.70—0.80, while six subscales had values between 0.50-0.60. Three subscales had low reliability values that ranged between 0.28-0.49. We note that all three of these subscales with low reliabilities were those with only two items, and that a low number of items in subscales can affect Guttman’s Lambda values [49]. Due to the exploratory nature of this study, the theoretical importance of the subscale constructs, and the low sample size (*N* = 81), a decision was made to retain these subscales for preliminary analysis, with the understanding that a later study involving a larger sample size (i.e., N > 300) will allow for a factor analysis to determine if these subscales and associated items need to be omitted.

**Table 5:** Means, standard deviations and Guttman’s Lambda values for the ARRQ subscales

Measure	N	*M*	*SD*	Λ
Self-esteem (2 items)	79	6.53	2.07	.59
Persistence (2 items)	78	6.81	1.92	.28
Meaning (2 items)	79	7.91	1.98	.72
Trust (2 items)	80	6.21	1.97	.59
Self-awareness (2 items)	79	7.72	1.75	.56
Self-responsibility (2 items)	79	8.15	1.73	.42
Emotional regulation (3 items)	79	9.46	3.21	.69
Positive emotions (4 items)	80	14.10	3.23	.65
Role models/mentors (2 items)	80	6.91	2.26	.62
Attachment (2 items)	80	7.86	2.23	.75
Safety(5 items)	79	18.48	4.06	.63
Social support (4 items)	78	14.42	4.36	.81
Self-efficacy (2 items)	81	7.15	2.07	.77
Personal mastery (2 items)	80	7.48	1.94	.49
Communal mastery (2 items)	80	6.88	2.11	.70
Problem solving (2 items)	80	7.10	2.01	.79
Community connection (4 items)	79	15.75	3.00	.60
Community opportunity (3 items)	80	11.03	3.18	.81
Cultural identity (3 items)	79	14.25	1.40	.73

^*^N = number of participants who completed the subscale

### The structure of the ARRQ

The Kaiser-Meyer Olkin value was 0.84, which exceeded the recommended value of 0.6 [42] verifying the sampling adequacy for the analysis. Bartlett’s test of sphericity reached statistical significance, χ^2^ (171) = 665.82, *p* < 0.001, indicating that correlations between items were sufficiently large for the Principal Components Analysis. A Principal Components Analysis was conducted on the 19 subscales of the ARRQ with oblique rotation (Direct Obliman) to maximise difference between the components. An initial analysis was run to obtain eigenvalues for each component in the data. There were four components with eigenvalues exceeding one which together explained 62 per cent of the variance (38.4, 10.6, 7.2 and 5.7 per cent, respectively). An inspection of the scree plot revealed two points of inflection, suggesting retention of two or three components for the final analysis [49]. As illustrated in Table 6, the results of Horn’s [50] parallel analysis, conducted using O’ Connor’s [51] syntax for SPSS, indicated that only two components had eigenvalues that were larger than those obtained from a randomly generated data matrix of the same size. Therefore, two components were extracted.

**Table 6:** Eigenvalues from principal component analysis and criterion values from parallel analysis

Component number	Raw data Eigenvalue	Criterion mean value parallel analysis	Decision
1	7.29	1.96	Accept
2	2.02	1.83	Accept
3	1.36	1.66	Reject
4	1.07	1.52	Reject

Examination of the subscales with large component loadings suggests that Component 1 assessed *Personal Strengths*, whereas Component 2 assessed *Relational-Community-Cultural Strengths*. Both components with respective subscale loadings are illustrated in Table 7*.* The two-component solution accounted for 49 per cent of the variance in responses, with Component 1 accounting for 38 per cent and Component 2 accounting for 10 per cent. The components were moderately, positively correlated (*r* = 0.58, *p* < 0.01).

**Table 7:** Pattern matrix for the 2-component solution of the ARRQ

Subscale	Component 1 Personal Strengths	Component 2 Relational-Community Cultural Strengths
Self-responsibility	.80	
Self-efficacy	.79	
Persistence	.79	
Problem solving	.78	
Self-awareness	.76	
Self-mastery	.73	
Self-esteem	.66	
Trust	.57	
Positive emotion	.56	.32
Community connection	.43	
Emotion regulation	.40	.32
Meaning	.40	
Attachment		.95
Safety		.91
Social support		.90
Role models		.70
Communal mastery		.60
Community opportunity		.40
Cultural identity		.34

Table 7 shows loadings >.30

### Preliminary examination of convergent and discriminant validity

Pearson correlation coefficients were used to assess the convergent and discriminant validity of the ARRQ. As presented in Table 8, all three strength scores on the ARRQ – that is, the personal strengths component, relational-community-cultural strengths component, and the total strengths scores, showed significant strong negative correlations with trauma symptom severity, and strong positive correlation with empowerment. Taken together, these correlations provided preliminary evidence that the ARRQ has good convergent and discriminant validity.

**Table 8:** Pearson correlations

Variables	1	2	3	4
1 Total ARRQ strengths				
2 Relational-Community-Cultural strengths	.86^**^			
3 Personal strengths	.92^**^	.60^**^		
4 Growth and Empowerment	.68^**^	.55^**^	.67^**^	
5 Trauma symptom severity	-.56^**^	-.54^**^	-.50^**^	-.47^**^

^*^p < .05

^**^p < .01

## Discussion

The qualitative findings from the first focus group interview documented some important understandings about resilience, healing, and recovery from trauma, based upon the perspectives of six Victorian Aboriginal health professionals from an Aboriginal community-controlled counselling service. These insights shaped the development of the ARRQ. The participants identified a wide range of strength and protective factors deemed integral to processes of healing and recovery. They reviewed and constructed items so that the language and meaning of each item would be acceptable for use in the Melbourne Victorian Aboriginal and Torres Strait Islander community. Participants viewed healing and recovery from trauma as a complex and uniquely personal journey, and one that potentially involved many factors and different pathways. They identified resilience, healing and recovery, and post-traumatic growth as all potentially important processes or pathways with regards to how Aboriginal and Torres Strait Islander help-seeking clients responded to potentially traumatic events. These processes were not seen as entirely distinct from each other and could fluctuate and co-occur in people’s lives and journeys. This understanding is consistent with observations and findings from the indigenous and non-indigenous literature on resilience and recovery from trauma. Milroy (cited in Mackean [52] p. 522) for example, made a qualitative distinction between healing and recovery, writing that ‘healing is not just about recovering what has been lost or repairing what has been broken…. it is about renewal’. Longitudinal studies involving non-indigenous populations have investigated post-trauma pathways and confirmed differences between resilience and recovery trajectories [53, 54].

The focus group expressed strong views that individual pathways needed to be understood and located within broader family, community, and societal structures, echoing the broad consensus across Aboriginal and Torres Strait Islander family violence reports that the magnitude of trauma experienced in some communities requires multi-level responses [48, 55, 56]. For example, an analysis of the recommendations from major federal and governmental reports on Aboriginal and Torres Strait Islander family violence conducted by Cripps [57] found recurring themes around the need for systems and structural level change. Changes cited with respect to improving the response to family violence included: increased access to resources and service delivery; community capacity building in the areas of programs, education and training for the prevention and healing of trauma; restoration of self-determination and community governance; and land rights. These broader structural changes are consistent with the idea that for large-scale trauma, social and structural level responses precede individual trauma recovery and, indeed, are the essential factors in creating environments where healing and recovery from trauma is possible [54, 58].

Focus group participants agreed that no single factor could predict the healing process for an individual. Healing and recovery from trauma is likely to unfold uniquely according to individual differences in their experiences of trauma, resilience, and healing- and recovery-related factors. The Canadian Aboriginal Healing Foundation reported similar conclusions after evaluating the organisation’s first seven-year cycle of funding community-designed healing programs across First Nations groups in Canada [59–61]. The organisation found that while healing processes and programs included some common phases and elements, their evaluation also identified that personal, family, and community history factors varied considerably between individuals participating in healing programs (60, 61]. However, the evaluation was unable to specifically examine individual differences in such factors due to the high levels of program diversity. The results from this psychometric analysis of the preliminary version of the Aboriginal Resilience and Recovery Questionnaire (ARRQ) are consistent with the organisation’s evaluation findings, as there was substantial variation in strength scores reported by participants using the ARRQ.

The two components of personal strengths and relational-community-cultural strengths as sources of resilience and recovery share some similarities with other resilience measures developed in Western populations, such as the Connor Davidson Resilience Scale [62] and the Resilience Scale for Adults [63] both of which include questions about coping with stress for example. Where the ARRQ differs to these resilience measures is that almost a quarter of the items (*n* = 13/60) constructed and endorsed by participants were linked to culture and community, including constructs such as cultural identity, cultural practices, community connection, and perceived opportunity and support in community. These protective factors identified by the focus group align with Kickett’s [7] definition of resilience that recognises the importance of belonging and connection to family and community. Specific items in the ARRQ that focus on cultural identity and values also share some similarity with items in the Westerman Aboriginal Symptom Checklist’s ‘Cultural Resilience subscale’ [29]. One of the strengths of the ARRQ, whilst noting this study represents a preliminary analysis only, is that it consists of multiple dimensions of resilience in the form of subscales and components that were developed based on Aboriginal practitioner experiences and allows for assessment of individual differences in different types of strengths and protective factors.

The Child and Youth Resilience Measure (CYRM), developed by Ungar and colleagues [19, 23], similarly assesses multiple strengths components. Perhaps not surprisingly, of the measures located to draft ARRQ items, this was the one most utilised due its emphasis on access to resources and opportunities in community. Although specific comparisons between this preliminary investigation of the psychometric properties of the ARRQ and that of the CYRM are not possible, we note some broad congruency in our findings and that of research reviewed in the introduction. For example, the early validation study 28 item CYRM conducted by Lienbenberg, Ungar and Fons Vab de Vijver [23] found that personal and relational strengths loaded on different subscales. Similarly, the two principal component factors of the ARRQ differentiate between individual strengths and resources (i.e., personal strengths) and external-oriented strengths and resources (i.e., relationship-community-cultural). This distinction between individual versus relationships, community and cultural dimensions of resilience has been replicated in other validation studies involving the CYRM [64]. In their study involving indigenous students in the Northern Territory, Robinson and colleagues [21] found that the 12 item CYRM socio-cultural resilience subscale was associated with lower psychological distress. Similarly, we also found a relationship between cultural dimensions of resilience and a distress-related construct, with relationship-community-cultural subscale scores predicting lower trauma symptom severity among Aboriginal help-seeking clients. We note again the different populations and cultural contexts of these studies (i.e., youth versus adult, and education versus help-seeking contexts), and importantly we acknowledge that the limited sample size of our preliminary study precluded conducting exploratory and confirmatory factor analyses, (which was used in the studies described above).

The preliminary results detailing the psychometric properties of the ARRQ are encouraging for several reasons. First, the correlation between the personal strengths and relational-community-cultural strengths subscales is moderate (0.6), suggesting that these subscales represent distinct but related types of resilience resources. Second, both subscale scores and the ARRQ composite strength scores demonstrated convergent and discriminant validity. Specifically, both subscales and the total strengths scores demonstrated moderate to strong positive associations with empowerment, and moderate to strong negative associations with trauma symptom severity. To our knowledge, this is one of only a few strength-based quantitative measures developed with indigenous populations that is specifically designed for use in research that has demonstrated a relationship between cultural determinants/strengths and trauma symptom severity. The strength of the ARRQ is that it provides a comprehensive range of individual, relational, community and cultural resilience factors that experienced local Aboriginal health professionals viewed as central to enabling clients to heal and recover from trauma.

### Limitations

However, there are some significant limitations with respect to psychometric findings from Study two that indicate quite clearly the need for further investigation of the items, and reinforce the understanding that this study needs to be viewed as preliminary in nature. Three of the 19 subscales (Persistence, Self-Responsibility and Personal Mastery) had low reliability values, which indicates that the items within the subscale lack homogeneity and do not at this stage represent the specific resilience construct well. We note here that these subscales were limited to two items which does influence reliability values. At this stage of measure development, the importance of these constructs for focus group participants, and the small sample size in this preliminary study, dictated that these subscales and items were retained. However, future research with a larger sample size will allow for more sophisticated forms of statistical analysis such as factor analysis (e.g., exploratory and confirmatory factor analysis). These techniques require a minimum of 300 observations to allow for greater computation power [35, 36] and would allow for all 60 items to be factor analysed and help determine whether some items should be dropped. Following the study reported in this article, our team is in the process of combining data sets to reach a minimum number of 300 participant ARRQ scores required for a more sophisticated factor analysis of the ARRQ measure.

There is also some conceptual overlap in the two components of the ARRQ. For example, it is not clear why the Community Connection subscale loaded onto the personal strength component and not the relational-community-cultural component. In addition, there was some evidence of cross loading for the Positive Emotion and the Emotional Regulation subscales. A common factor analysis involving entering all 60 ARRQ items separately into the analysis (requiring 300 participants) may assist in resolving this issue and help determine whether the multifactorial structure of the measure is stable.

### Future directions

Beyond the psychometric limitations stated in this preliminary study documenting development of the ARRQ, an important question is how could the strength-based ARRQ be utilised in future research and service delivery to support Aboriginal help-seeking clients healing, recovery and wellbeing? There are several fruitful avenues that could be considered. One is to investigate the potential buffering role that strengths outlined in the ARRQ may have with regards to the relationship between trauma exposure and trauma-related distress among help-seeking clients. A second, related issue, is if the strengths outlined in the ARRQ did confer protective effects with regards to experiencing stress, trauma, or other adversity, can programs be developed in the Aboriginal community-controlled health sector (and other services and sectors) that support help-seeking clients to build these strengths? These could be recovery related programs, although building these types of strengths could also play a prevention role with regards to wellbeing outcomes. Longitudinal and prospective studies located in service programs that target prevention of family violence, reduction of trauma related distress, and/or building coping skills are what may be required to fully answer these questions. However, a stepwise approach may be required beginning with cross-sectional studies investigating the relationships between adversity, strengths, and distress, as well as studies investigating whether the ARRQ is sensitive to change. These kinds of initiatives have progressed since development of the ARRQ, though further research is required [65, 66].

Finally, we recognise that in its current form, 60-items poses considerable participant burden for those filling out the measure. It will be valuable for future research to investigate the potential for a short form version that performs adequately without sacrificing the established adequate psychometric properties of the ARRQ.

## Conclusion

At this stage of development, our research indicates that within an urban Aboriginal Victorian context the preliminary version of the ARRQ holds potential as a culturally valid strengths measure. It was developed from Koori and Aboriginal Victorian understandings of resilience, healing, and recovery from trauma, and could be suitable for use in quantitative research. The ARRQ has the potential to assist Aboriginal Community Controlled Health Organisations and, indeed, other organisations to evaluate whether their services and programs are supporting community members to strengthen individual, relational, community and cultural resilience resources.

### Supplementary Information

**Additional file 1.**

## Data Availability

The data that support the findings of this study are available from the Victorian Aboriginal Health Service but restrictions apply to the availability of these data, and so are not publicly available. Deidentified data are available from the corresponding first author (GG) upon reasonable request and with permission of the Victorian Aboriginal Health Service.

## Abbreviations

ARRQ: Aboriginal Resilience and Recovery Questionnaire
VAHS: Victorian Aboriginal Health Service
FCS: Family Counselling Services
CYRM: Child and Youth Resilience Measure

## Footnotes

1 This article uses the term ‘Aboriginal and Torres Strait Islander to respectfully refer to the diversity of First Peoples in Australia, the term ‘indigenous’ and ‘First Peoples’ for broad reference to First Peoples worldwide, and specific terms for different First Peoples when citing literature that references accordingly. These terms are used deliberately to reflect the preferences held by many Aboriginal and Torres Strait Islander peoples and other respective indigenous First Peoples worldwide.

2 The term ‘*Koori*’ (meaning ‘people’ in Eastern and Western Kulin dialect) is used to refer specifically to any Aboriginal person or peoples that identify as belonging to one or more of the traditional Aboriginal language or clan groups located in Victoria, Australia. The term ‘Aboriginal Victorians’ will be used more broadly to refer to all Aboriginal peoples living in Victoria.
