# Supplementary material for: Development of an Aboriginal Resilience and Recovery Questionnaire – a collaboration between practitioners and help-seeking clients of a Victorian Aboriginal community controlled health service
Source: BMC Med Res Methodol. 2023 Dec 8;23:290. doi: 10.1186/s12874-023-02091-4 (PMC10709844; doi:10.1186/s12874-023-02091-4)
Supplement: Supplementary file 1 — Additional file 1. [file 12874_2023_2091_MOESM1_ESM.docx]

**Supplementary File 1**

Appendix - Preliminary Aboriginal Resilience and Recovery Questionnaire items (Gee, 2016)

I am proud to be Aboriginal or Torres Strait Islander

Being Aboriginal or Torres Strait Islander is an important part of who I am

I am able to maintain my Aboriginal or Torres Strait Islander identity, values and beliefs

In my community I have opportunities to develop skills (e.g., job skills or skills to care for others)

In my community I have opportunities to further my education

I have opportunities to work in my life, keep busy and stay involved

I am able to overcome most of my problems by working together with my friends and family

What happens to me in the future depends most of all on the support of my friends and family

I feel supported by my friends/mob

I can talk about my problems with family or friends

I have family that love me even when I muck up

In my family we can talk with each other about most things

In my everyday life I have role models that I look up to

In my everyday life I have people who listen to me and believe in me

There are people in my life that I have close, secure relationships with

I can turn to my partner or someone close to me for support and understanding

I feel safe when I am with my partner or those closest to me

I feel safe when I am with my family

I feel safe in my community

I feel safe in the broader society outside my community

I have a safe place to go to where I can heal

I feel pride in my achievements

I am ok with myself as I am now

When I experience setbacks I don’t give up

I usually finish what I start

There is meaning in what I do in my daily life

I have things in my life that I’m passionate about

I can trust myself to make the right choice

Despite any bad experiences in the past I am able to trust most people

I am aware of my strengths and weaknesses

I try to understand why things happen to me

I choose not to blame other people for the decisions I make

I am responsible for my own happiness

I can talk about how I feel when I get upset

I am able to face problems without gambling, using drugs or alcohol, or harming others

I can handle painful or upsetting emotions such as anger and sadness

On a daily basis things happen that make me happy

I feel content with my life

I feel compassion for the pain others feel

I am able to forgive myself and others

I am able to deal with most problems that occur in my life

When changes occur in my life I can usually find ways to adapt

Overall, I feel like I have control over my life

What happens to me in the future depends most of all on me

I take positive action to try and solve problems

When I have a problem I make plans about how to deal with it

I spend time helping others in my community

I know where to go in my community for help

I feel like I belong in my community

I am treated fairly in my community

I participate in cultural practices that give me peace (such as going out bush, ceremony, community cultural events)

Spirituality is a source of strength for me

When stressed I am able to take time to care for myself (e.g., time alone, relaxation)

I am able to accept difficult things that have happened in my past

I have opportunities to tell my story and make sense of things that have happened to me

I am able to have a laugh even when things are difficult

I use art, music, sports or similar activities as a way to express myself

I have the skills to be confident in both Indigenous and non-Indigenous communities

I find it easy to get along well with people

I feel confident in socialising with others around me
